# Anomalous Origin of the Left Coronary Artery From the Pulmonary Artery: An Uncommon Coronary Anomaly With Serious Implications in Adulthood

**DOI:** 10.1177/2324709616684629

**Published:** 2017-01-01

**Authors:** Venkat Gangadharan, Kamesh Sivagnanam, Ghulam Murtaza, Michael Ponders, Otto Teixeira, Timir Paul

**Affiliations:** 1 Department of Internal Medicine, Division of Cardiology, East Tennessee State University, Johnson City, TN, USA; 2 Department of Internal Medicine, East Tennessee State University, Johnson City, TN, USA; 3 Department of Paediatric Cardiology, East Tennessee State University, Johnson City, TN, USA

**Keywords:** anomalous coronary artery, coronary anomaly, congenital heart disease

## Abstract

A 36-year-old woman was seen with complaints of exertional chest pain and shortness of breath. Her medical history included atrial fibrillation and diabetes. Physical examination was unremarkable except for an irregular cardiac rhythm. Myocardial perfusion imaging revealed the presence of a large area of infarction involving the entire anterior and apical walls and part of the anteroseptal wall with minimal periinfarct ischemia. Computed tomography coronary angiogram revealed an anomalous left main coronary artery arising from the main pulmonary artery. Right and left heart catheterizations demonstrated moderate pulmonary hypertension with a slight step-up in oxygen saturation between the right ventricle and main pulmonary artery. Coronary angiography showed a large tortuous right coronary artery with collaterals to the left anterior descending artery that drained into the main pulmonary artery. She was referred for surgery. This case demonstrates a rare coronary artery anomaly in an adult where survival is dependent on collateral circulation.

## Introduction

Anomalous origin of the left coronary artery from the pulmonary artery (ALCAPA) is a rare congenital cardiac defect with an incidence of 1 in 300 000 live births.^1^ Most patients survive fetal life. However, as the pulmonary artery resistance falls after birth, there is a “steal” of blood flow from the myocardium to the pulmonary artery in the direction of the pressure gradient.^2^ This results in volume overload of the left-sided chambers, early heart failure, and myocardial ischemia.^2^ Symptoms begin by 2 weeks of age. Life may extend into the neonatal period; however, survival to adulthood is rare.

## Case Report

**Figure 1.**
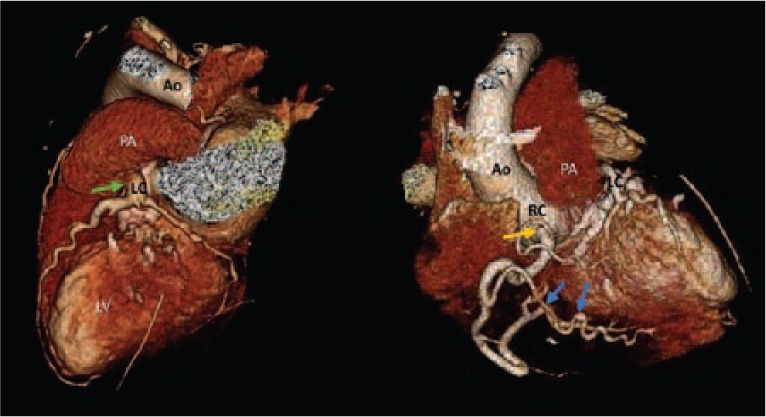
Three-dimensional rendering of coronary CT angiogram demonstrating origin of the right coronary artery (RC) which originates in the aorta (Ao) and provides collaterals across the left ventricle (LV) to supply the left coronary artery (LC) that originates in the pulmonary artery (PA).

**Figure 2.**
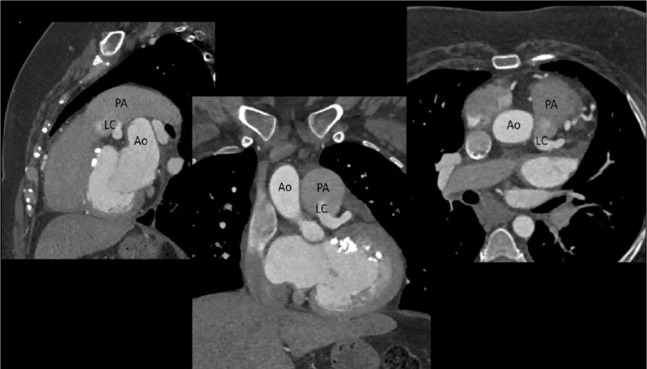
Sagittal, coronal, and transverse CT slices showing the origin of the left coronary artery (LC) from the pulmonary artery (PA). The aorta (Ao) is shown in perspective.

**Figure 3.**
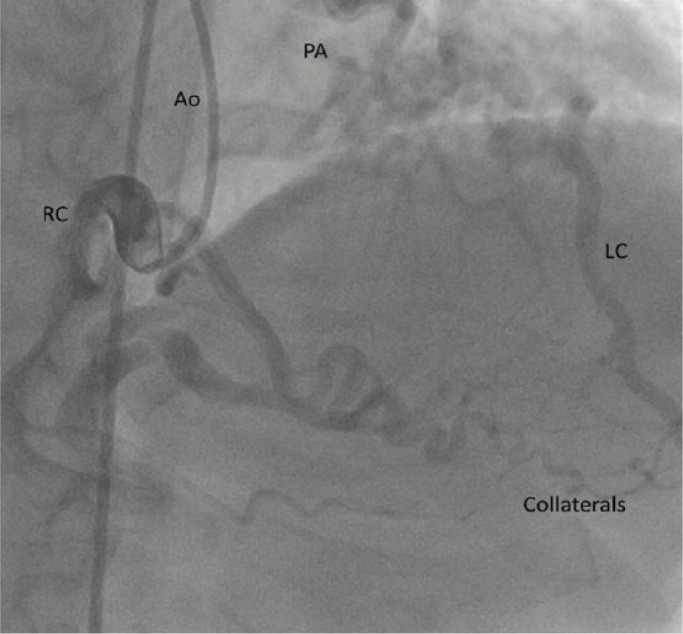
Coronary angiogram demonstrating large tortuous right coronary artery (RC) with collaterals to distal LAD (LC) and filling of LAD in a retrograde fashion. The relative positions of the aorta (Ao) and the pulmonary artery (PA) are also seen.

**Figure 4.**
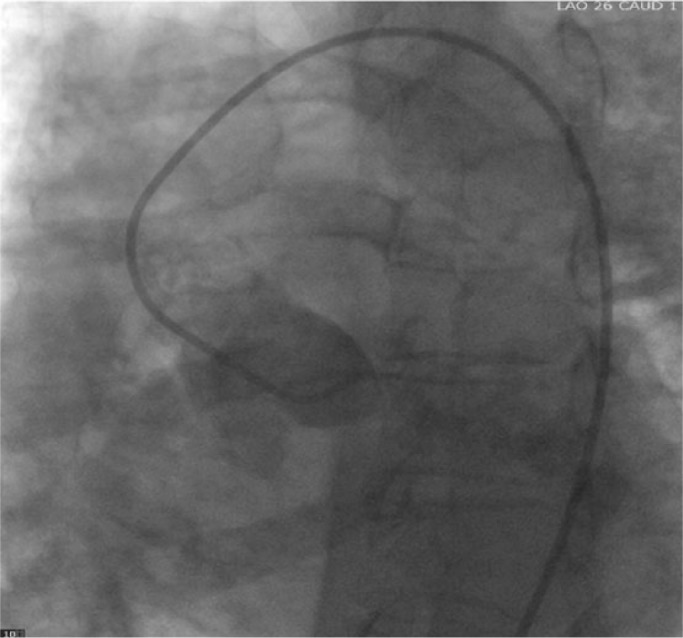
Nonselective injection into the left coronary cusp demonstrating the absence of the origin of the left coronary artery.

A 36-year-old female was referred to a cardiology clinic following an abnormal treadmill stress test. She had frequent episodes of exertional chest pain and shortness of breath with no palpitations, dizziness, or syncope. She had a history of atrial fibrillation, type 2 diabetes mellitus, and obstructive sleep apnea. Her medications included aspirin, coumadin, furosemide, losartan, metformin, simvastatin, spironolactone, and vitamin D_2_. Her physical examination was unremarkable except for irregularly irregular rhythm. An electrocardiogram showed atrial fibrillation with nonspecific ST-T wave changes. Myocardial perfusion imaging revealed a large area of infarction involving the entire anterior and apical walls and part of the anteroseptal wall with minimal periinfarct ischemia. The estimated ejection fraction (EF) was 36%. Subsequent 2-dimensional (2D) echocardiogram revealed an estimated EF of 35% to 40% with severe hypokinesis of the apex and inferior septum, moderate to severe dilatation of left atrium, severe mitral subvalvular calcification with no stenosis, and mild regurgitation. Coronary computed tomography (CT) angiogram revealed an anomalous origin of the left coronary artery system from the main pulmonary artery (Figures 1 and 2). A right heart catheterization was performed, which showed a small (<8%) step-up of oxygen saturation from the right ventricle to main pulmonary artery. The main pulmonary artery, right ventricle, and right atrial pressures were moderately elevated. Selective coronary angiograms revealed a large right coronary artery (RCA) with significant collaterals from the proximal RCA and distal posterior descending artery to the distal left anterior descending (LAD) artery, filling in a retrograde fashion (Figure 3). The left coronary artery appeared to drain into the proximal right pulmonary artery. The absence of a left coronary artery system arising from the left coronary sinus was confirmed with injection of contrast into the left coronary cusp (Figure 4). Given her ongoing symptoms, the patient was referred for surgical correction of her anomalous coronaries. At 6-month follow-up, her EF had significantly improved to 57%.

## Discussion

ALCAPA was first described in 1933 by Bland, White, and Garland in a 3-month-old child who died because of this anomaly.^3^ Anomalous left main coronary artery, LAD, or left circumflex arteries arising from the main or right pulmonary artery are uncommon, occurring in 0.26% of patients undergoing cardiac catheterization.^4^ Presentation in adulthood is extremely rare, and survival is heavily dependent on collaterals from the RCA that develop over several years.^5^

High pulmonary pressures at birth ensure adequate perfusion to the ventricular myocardium supplied by the anomalous coronary circulation. With time, as the physiologic decrease in pulmonary artery resistance and pressure occurs, blood flow through the anomalous coronary is compromised. This results in ischemia, impaired left ventricular function, and increased left ventricular end-diastolic pressure. This ischemic state in the distribution of the left coronary artery stimulates the development of collaterals from the RCA, which are pivotal for survival.^1^

Clinical manifestations may range from severe congestive heart failure and recurrent myocardial ischemic events in early infancy to nonspecific symptoms seen more often in adolescents and adults.^6^ The lack of adequate collateral supply from the right coronary system may result in subendocardial ischemia and ventricular arrhythmias, which are the leading causes of death in the adult population.^7^

In adults, coronary artery origins are difficult to visualize on routine echocardiography, requiring other noninvasive modalities to aid the diagnosis. Multidetector CT angiography allows for excellent visualization of the coronary arteries but lacks the ability to assess coronary flow, valvular function, and myocardial viability, which is better assessed using cardiac magnetic resonance imaging.^8^ Definitive diagnosis, however, is achieved with coronary angiography, which reveals both the presence of epicardial collaterals as well as the connection of the left coronary artery to the pulmonary artery.^8^

Surgery is the definitive treatment in neonates and corrects the system to resemble the normal coronary circulation. In adults, the decision to perform surgery depends on several factors including the presence of symptoms and evidence of significant disease. Chronic subendocardial ischemia or significant infarction is key indicator for surgery, with possible recovery of ventricular function and reduction in the risks of malignant arrhythmias.^9^ The surgery typically consists of either ligation of the left coronary artery or placement of a coronary artery bypass graft to the left system.^10^

## Conclusion

ALCAPA is a rare coronary anomaly in the adult population. Development of collaterals from the RCA supplying the left coronary system enables survival in this setting. Chronic subendocardial ischemia or infarction are indications for surgery in adults to improve symptoms and reduce the risks of malignant and often fatal arrhythmias.
